# Flexible learning spaces facilitate interaction, collaboration and behavioural engagement in secondary school

**DOI:** 10.1371/journal.pone.0223607

**Published:** 2019-10-04

**Authors:** Katharina E. Kariippanon, Dylan P. Cliff, Sarah J. Lancaster, Anthony D. Okely, Anne-Maree Parrish

**Affiliations:** 1 Early Start, School of Health and Society, Faculty of Social Sciences, University of Wollongong, Wollongong, NSW, Australia; 2 Early Start, School of Education, Faculty of Social Sciences, University of Wollongong, Wollongong, NSW, Australia; 3 Illawarra Health and Medical Research Institute, University of Wollongong, Wollongong, NSW, Australia; University of Kentucky, UNITED STATES

## Abstract

Globally, many schools are replacing traditional classrooms with innovative flexible learning spaces to improve academic outcomes. Little is known about the effect on classroom behaviour. Students from nine secondary schools (n = 60, M age = 13.2±1.0y) were observed via momentary time sampling for a 30 minute period, in both a traditionally furnished and arranged classroom and a flexible learning space containing a variety of furniture options to accommodate different pedagogical approaches and learning styles. The teaching approaches in both conditions were documented. In traditional classrooms the approach was predominantly teacher-led and in the flexible learning space it was student-centred. Students in flexible learning spaces spent significantly more time in large group settings (d = 0.61, *p* = 0.001), collaborating (d = 1.33, *p* = 0.001), interacting with peers (d = 0.88, *p* = 0.001) and actively engaged (d = 0.50, *p* = 0.001) than students in traditional classrooms. Students also spent significantly less class time being taught in a whole class setting (d = -0.65, *p* = 0.001), engaged in teacher-led instruction (d = -0.75, *p* = 0.001), working individually (d = -0.79, *p* = 0.001), verbally off-task (d = -0.44, *p* = 0.016), and using technology (d = -0.26, *p* = 0.022) than in traditional classrooms. The results suggest that the varied, adaptable nature of flexible learning spaces coupled with the use of student-centred pedagogies, facilitated a higher proportion of class time interacting, collaborating and engaging with the lesson content. This may translate into beneficial learning outcomes in the long-term.

## Introduction

Internationally, the education sector is undergoing a paradigm shift that encompasses both innovative built learning environments and significant reform of the pedagogical core [1,2], to better prepare students across all curriculum areas and learning stages to succeed in a rapidly changing and interconnected world [3,4]. An array of learning environments are emerging across educational institutions as educators strive to adapt their teaching practices and enhance learning outcomes [5]. These ‘flexible learning spaces’ contain a variety of furniture options in a relatively open space, which can be configured in various ways to facilitate a range of teaching and learning experiences [6]. They stand in stark contrast to traditional classrooms which are characterized by rows of desks and chairs, facing a teacher at the front who employs predominantly didactic teaching approaches. These traditional environments are now considered inadequate to deliver 21st-century competencies for learners [7–10]. The shift in practice is mirrored by an emerging research focus on the spatiality of education [11].

In Australia, in parallel to the shift underway in the education sector, a significant policy initiative Building the Education Revolution (BER), was launched as part of the Federal Government’s response to the global financial crisis. This resulted in funding managed at a State/Territory level, for schools to develop new learning spaces [12]. The initial investment has been followed by further financial commitments, and broad support at the departmental level for schools to transition to more flexible learning spaces.

The vision for future-focused learning environments is that learning will be enhanced through increasingly employing student-centred pedagogies. In the context of the schools participating in this study, this umbrella term incorporates project-based and personalised learning experiences that support deeper investigation into areas of personal interest beyond what is delivered to the whole class [5]. Further, these approaches enable students to be engaged as co-creators of the learning experience, both independently and collaboratively. This is much like the secondary education reforms in the Netherlands where a shift is occurring from learning environments based on knowledge transmission to those designed for knowledge construction [13]. Further the spaces and how they are used, facilitate ample opportunities to enhance student creativity, innovation, communication and problem solving skills, which are deemed increasingly crucial for the workplaces of the future that students are being prepared for by schools. These learning environments ideally support student choice of where and how to learn and enable easy access to a range of educational technologies designed to facilitate learning [6]. The incorporation of virtual space into learning environments necessitates additional modifications to both the built environment and the pedagogical approach to capitalise on the affordances of technology [14].

It is purported that flexible learning spaces inherently support educators to employ student-centred teaching approaches [15], and that these spaces can accommodate and facilitate learning modes such as collaboration, explicit instruction, independent work, feedback and reflection as well as experiential learning, which are believed to lead to improvements in students’ engagement and motivation [16]. In turn, the cognitive, social and behavioural domains of student engagement are collectively associated with improved learning outcomes such as retention of knowledge, test scores and grades [17]. It is broadly assumed that the teaching and learning approach used in flexible learning spaces will ultimately lead to improvements in academic outcomes.

Although an estimated 25% of Australian classrooms are now no longer classified as ‘traditional’ [18], there is limited empirical evidence on the effects that flexible learning spaces have on adolescent classroom behaviour and ultimately learning outcomes in the secondary school setting [19,20]. Despite the dearth of evidence, significant funds are being invested across Australia at Federal and State levels to both refurbish existing classrooms and fit out new builds [21]. Changes to the built environment are increasingly accompanied by an array of professional development opportunities for teachers. However, limited inter-disciplinary research exists that draws on learnings from the built environment literature and current understanding of school improvement and educational change processes, to ensure that teachers are effectively prepared and supported to transition to flexible learning spaces [19].

School educators are now faced with the challenge of navigating evolving teaching landscapes in these innovative environments, are required to adopt a flexible and adaptive pedagogical approach and provide increasingly personalised support to students. However, previous research has shown that regardless of improvements in spatial configuration, physical features or classroom furnishing, direct instruction remains the dominant pedagogical approach used in schools [22], highlighting that pedagogical adaptation is not necessarily a natural flow-on from changes to the built environment. This may be attributed to teachers’ environmental competence, with many teachers lacking the ability to manipulate the learning environment to capitalize on the affordances of the space to maximise pedagogical gain [23]. In addition, the structure of space within buildings is thought to influence the formation of relationships between people [24], yet little is known about the nature of interactions that occur with these spaces.

While acceptability of flexible learning environments is relatively high, and teachers and students report perceived benefits to teaching, learning and wellbeing [5], few studies have observed flexible learning spaces in action or have systematically documented student behaviour to determine the impact that flexibility of space and mobility of technology and furniture have on space use [19]. Effective design of leaning spaces has been found to facilitate constructivist pedagogy and student engagement [25,26] and research suggests that how classroom space is arranged has implications for student performance [27]. However, the modes of learning students engage in, the physical settings they choose, how they interact with their teachers and peers, and the effect of these innovative environments on behavioural engagement remain largely unexplored [28]. The aim of this study was therefore to objectively measure and compare adolescent classroom behaviour between traditional classrooms and flexible learning spaces and assess the effect of the space and teaching approach on a range of classroom behaviours.

## Methods

The protocol was approved by the University of Wollongong’s Human Ethics Research Committee (HE16/021) and the New South Wales (NSW) State Education Research Applications Process (SERAP).

### Participants

Purposive sampling was used to identify schools that had created at least one flexible learning space within their school, which students used on a regular basis. Changes included modifications to both the physical environment and the pedagogical approaches used in the space. Invited schools had made these changes prior to the launch of the funding initiative by the NSW Education Department, often with limited resources, and prior to this study (independent of the researchers). The study was a school-based cross-over trial, with Grade Seven-Nine classes from 12 public schools in NSW Australia, invited to participate. Informed parental consent was obtained and data collection included the students’ age, sex, cultural background and postcode of residence, which was used to determine socioeconomic status.

### Learning space conditions

#### Traditional classrooms–built environment

**Fig 1:**
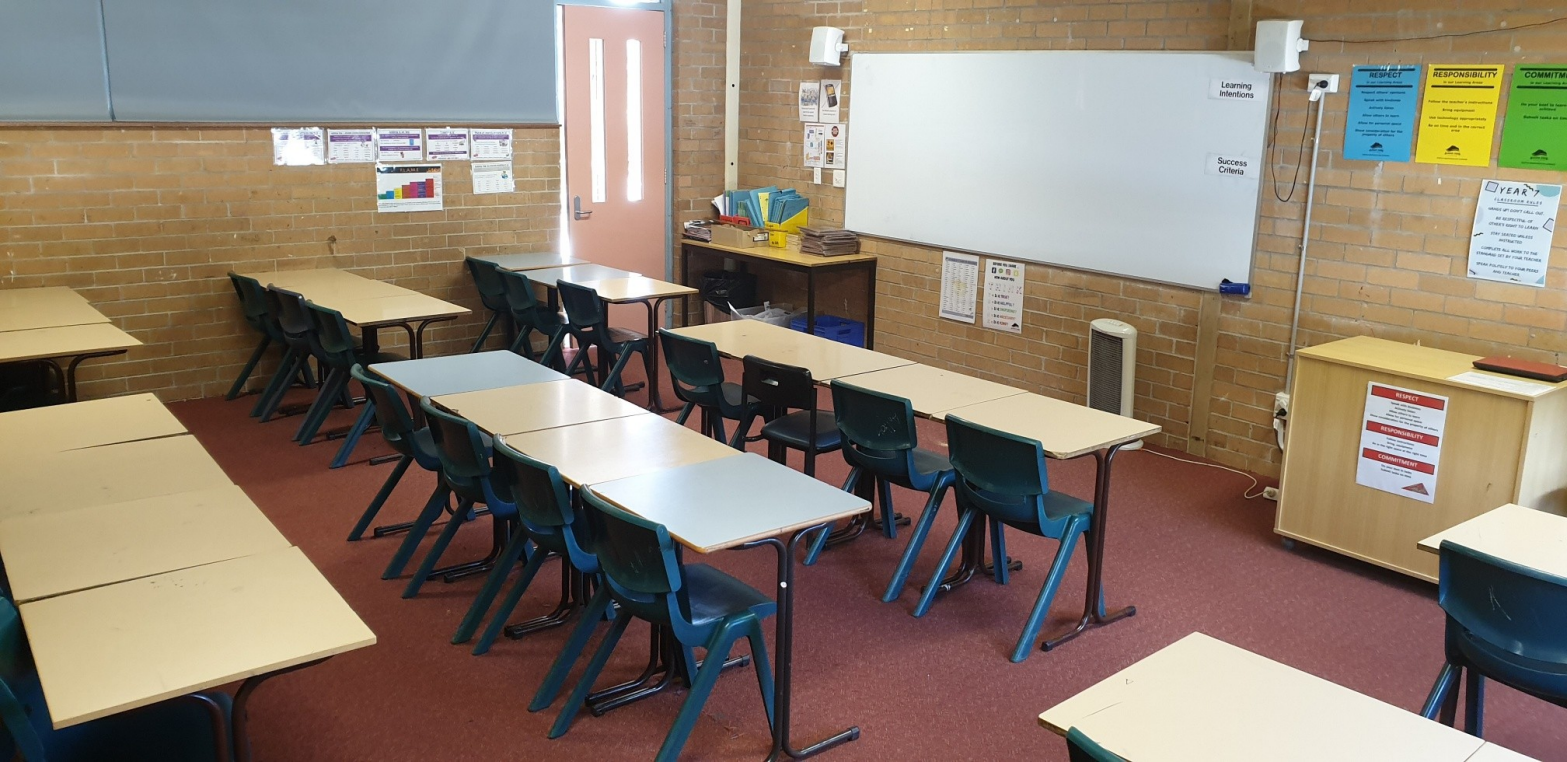
A traditional classroom.

Traditional classrooms (Fig 1) were a standard single classroom (M = 50m^2^), which typically contained a desk and chair for each student, arranged in rows of paired desks or a u-shape facing the front. Students chose their seat upon entering the classroom and generally remained there for the duration of the lesson.

#### Flexible learning spaces–built environment

**Fig 2:**
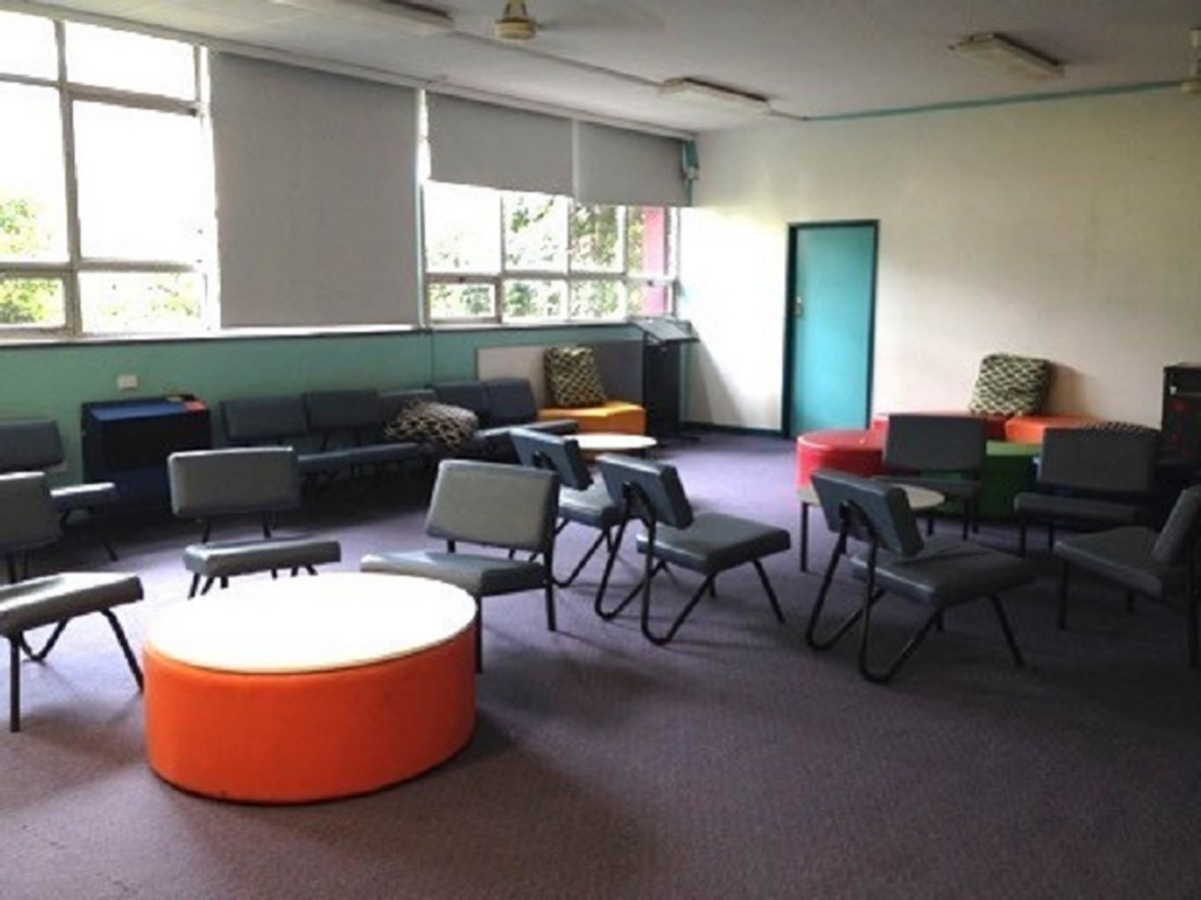
A flexible learning space.

Flexible learning spaces (Fig 2) were a combination of standard- and double-sized classrooms (M = 83m^2^) and incorporated a range of furniture such as grouped tables, standing workstations, ottomans, couches, and write-able tables and walls. The majority of flexible learning spaces lacked a distinct front of the classroom, with resources including smart boards and whiteboard walls available around the room, giving the teacher greater flexibility to move around the space.

### Teacher professional development

Prior to the study, teachers had participated in various professional learning experiences ranging from tours of other schools that had transitioned to flexible learning spaces, conferences, short courses on designing and teaching in flexible learning spaces and informal teacher networks, both within and among schools, that gave rise to collective reflexivity and reciprocal learning [5]. All participating teachers fully embraced the underlying principles of what a flexible learning space could enable and shared a common vision for providing students with a learning environment designed to enhanced learning. These teachers were considered change agents within their respective schools [5].

### Instrument

Students’ in-class behaviour was systematically observed using momentary time-sampling. The instrument was based on a previously validated observational tool, the *Classroom Observation System*, *COS-5 Pianta* [29], which aims to record the frequency of a range of behaviours and experiences that may typically be observed in a school classroom. In consultation with the research team, *child level setting*, *child academic behaviour*, and *child social behaviour* were deemed behavioural categories relevant to the aims of this study. To provide additional detail and to ensure the tool was able to capture elements of interests that exist within a flexible learning space, a further two categories, namely *mode of learning*, and *use of technology* were added to the instrument. Table 1 provides detail on the categories, codes, and descriptions used in this study. To maximise the validity and reliability of the observations one researcher completed all observations. The researcher received two hours of training in identifying and classifying the behavioural codes and undertook practice observations from video recordings of lessons and during classroom lessons prior to data collection to develop a consistent understanding of the categories and to become familiar with the procedure.

**Table 1:** Observational categories.

Category	Codes	Description
Student level setting	Whole class, groups of >6, groups of ≤ 6, individual	These codes refer to the setting in which the student is working
Mode of learning	Teacher-led instruction, working individually, collaborating, presentation-based, reflective, research-based	This category captures the different forms of learning the student may engage in–this list is not exhaustive
Academic behaviour	Actively engage, passively engaged, off-task verbal, off-task motor, off-task passive	These codes describe the intensity and level of the student’s involvement with the set academic task
Interaction with peers	Positive interaction, negative interaction, no interaction	Codes in this category capture the nature of the social interaction the student has with their peers
Interaction with teacher	Positive interaction, negative interaction, no interaction	Codes in this category capture the nature of the social interaction the student has with their teacher
Use of technology	Active use, passive use, no use	These codes describe the use of technology

### Procedure

Schools were required to timetable the same group of students into both a traditional classroom and on another day a flexible learning space, for the duration of a double lessons (M = 72min). Prior to the commencement of data collection in each respective learning environment, a discussion was held with the teacher about the pedagogical approach for the lesson, the structure, content and what activities would be occurring.

A class list featuring consenting students in alphabetical order of surname was used to identify students to be observed. For the first observation in each school, the first three female and male students on the list were selected; if any were absent the next student of that sex was chosen. At the second data collection time point, the same six students were again observed. If a previously observed student was absent, the next student of the same sex on the class list would be selected. Neither the students nor their teacher were aware of who had been selected for observation. The lesson then proceeded as planned.

The observation began approximately 10 minutes after the lesson commenced, once students had settled into the lesson. The observer used headphones to alert them to observe and categorize the six student’s behaviour at 30-second intervals, on a rotational basis over a 30 minute period. Each student was therefore observed 10 times over the course of the 30 minutes. This procedure has been found to be effective when seeking to describe students’ classroom behaviour [30].

The teachers were all familiar with teaching both in their schools’ traditional classrooms and flexible learning spaces and recognized how their teaching approaches varied between the two learning environments [5]. Teachers were required to teach in a manner typical of how they would normally conduct their lesson in the respective learning spaces. The students and teachers had all spent significant time teaching and learning in both traditional classrooms and their school’s flexible learning space and quickly adjusted to the distinct ways of working in the two different environments. The lesson content and teacher were consistent across both conditions. Observations were conducted in subject areas including English, mathematics, geography, and history.

The two data collection instances in each school took place within 1–2 weeks of one another, between 2016 and 2017. Schools determined which condition was assessed first. Participating teachers were aware of the broad categories of behaviour and experiences being observed but had not seen the tool itself.

### Statistical analysis

Ten observations were recorded for each of the six students over a 30 minute period in both conditions. Raw data were entered into an excel spreadsheet. For each of the six behavioural categories, the number of times each code within the category occurred was converted into a percentage, for each participant, for each observation period. Analyses were conducted in SPSS (Version 21) and STATA (Version 13). Data were analyzed using mixed-effect multi-linear regression to calculate the differences between traditional classrooms and flexible learning spaces for all codes for each behavioural category. The model analyzed the data for within-child differences. To account for clustering, schools were used as a random effect in the model. The mean differences in outcome variables between the two conditions were considered statistically significant at p < 0.05. To demonstrate the magnitude of the difference between the means of the two conditions, standardized effect sizes (Cohen’s *d*) were calculated from the means and standard deviations of the two conditions; using the traditional classroom as the denominator. Effect sizes of approximately 0.2, 0.5 and 0.8 were considered small, medium and large respectively [31].

## Results

A total of 243 students from nine participating schools were invited to participate in the study and 203 provided informed consent (83%). Eight schools were co-educational and one school was an all-boys school. Schools typically selected their top academic class of the year level to participate in the research. Of the 54 students who were selected to be observed in the two conditions in each of the nine schools, a total of six students were absent at the second data collection time point, so six additional students were selected. Valid data were therefore obtained from 60 students. Of the sample 45% were female, students had a mean age of 13.2 years (SD = 1.0), and were from a range of socio-economic backgrounds, representing over 13 cultural and ethnic groups (Table 2).

**Table 2:** Descriptive statistics of study participants.

Sample (n)	60
Age (M & SD)	13.2 (1.0)
Proportion female (n, %)	27 (45)
SEIFA (M & SD)	1013.31 (73.61)

Two quite distinct pedagogical approaches were evident in the two learning environments across the nine schools.

### Traditional classrooms–pedagogical approach

In the traditional classrooms the teaching style was primarily teacher-led, with the teacher largely remaining at the front of the classroom, often near the teacher’s desk. Students generally worked individually on set tasks and received frequent input and additional instruction from the teacher. Students had limited reasons or options to stand or move around the room, or find an alternative place to work throughout the lesson or to engage with one another.

### Flexible learning spaces–pedagogical approach

The teaching approach in the flexible learning spaces was student-centred and group-work focused. Students were given instructions from the teacher regarding the lesson plan and objectives at the commencement of the class, and further guidance throughout the lesson as needed. In addition students were afforded considerable freedom to choose how to go about their learning. Together with the furniture available, this teaching approach created opportunities and incentives for students to move throughout the lesson. Students were given the autonomy to choose where in the space to work, what furniture and resource to use and typically formed groups or worked independently out of their own volition.

### Classroom behaviours

Significant differences were observed among multiple codes of classroom behaviour in all but the *interaction with teacher* category. (Table 3).

**Table 3:** Difference in lesson time students spent engaged in outcome variables between the traditional classrooms and flexible learning space.

Outcomes As a proportion of lesson time (%)	Traditional Classroom (*M*, *95% CI*) (*n = 54)*	Flexible Learning Space (M, 95% CI) (n = 54)	Mean difference in change between spaces (M, 95% CI)	Effect size (Cohen *d*)	*P* value
**Lesson time spent in different *learning settings***					
Whole class	32.22 (18.03, 46.42)	9.81 (-4.38, 24.01)	-22.41 (-33.30, -11.51)	-0.65	**0.001**
Groups of > 6	0.00 (-6.25, 6.25)	7.03 (0.79, 13.28)	7.04 (2.26, 11.82)	0.46	**0.004**
Groups of ≤ 6	53.52 (41.06, 65.98)	77.78 (65.32–90.24)	24.26 (9.98, 38.53)	0.61	**0.001**
Individual	14.26 (5.81, 22.71)	5.37 (-3.08–13.82)	-8.89 (-17.64, -0.14)	-0.35	**0.046**
**Lesson time spent in different *modes of learning***					
Teacher-led instruction	30.74 (18.55, 42.93)	14.26 (2.07, 26.45)	-16.48 (-21.06, -11.90)	-0.75	**0.001**
Working individually	52.41 (36.02, 68.80)	28.70 (12.38, 45.09)	-23.70 (-30.36, -17.05)	-0.79	**0.001**
Collaborating	12.59 (-2.74, 27.92)	49.44 (34.11, 64.77)	36.85 (31.00, 42.70)	1.33	**0.001**
Presentation-based	0.00 (-3.05, 3.05)	4.26 (1.21, 7.31)	4.26 (6.11, 3.05)	0.65	**0.001**
Reflective learning	1.67 (-1.16, 4.49)	2.22 (-.060, 5.05)	0.56 (-0.61, 1.72)	0.11	0.351
Research-based	2.59 (-0.77, 5.95)	0.93 (-2.43, 4.29)	-1.67 (-3.37, 0.04)	-0.25	0.055
**Lesson time and *type of engagement***					
Actively engaged	56.93 (48.69, 65.18)	68.98 (60.73, 77.22)	12.05 (5.15, 18.94)	0.50	**0.001**
Passively engaged	18.70 (12.90, 24.51)	14.26 (8.46, 20.06)	-4.44 (-10.10, 1.21)	-0.27	0.123
Off-task—motor	6.46 (3.05, 9.86)	5.17 (1.77, 8.58	-1.28 (-4.07, 1.50)	-0.13	0.367
Off-task—verbal	12.26 (8.55, 15.96)	6.50 (2.79, 10.20)	-5.76 (-10.46, -1.07)	-0.44	**0.016**
Off-task—passive	5.61 (2.54, 8.68)	4.97 (1.90, 8.05)	-.64 (-3.73, 2.46)	-0.07	0.686
**Lesson time and *type of interaction with other students***					
Positive interaction	35.47 –(26.07, 44.87)	58.34 (48.95, 67.74)	22.87 (14.97, 30.77)	0.88	**0.001**
Negative interaction	0.34 (-0.44, 1.11)	0.92 (0.14, 1.69)	0.58 (-0.25, 1.41)	0.21	0.173
No interaction	62.51 (50.53, 74.49)	38.69 (26.71, 50.67)	-23.82 (-31.44, -16.19)	-0.85	**0.001**
**Lesson time and *type of interaction with the teacher***					
Positive interaction	20.00 (11.61, 28.39)	20.56 (11.61, 28.39)	0.56 (-4.22, 5.34)	0.03	0.820
Negative interaction	0.19 (-.07, 0.44)	0.00 (-0.25, 0.25)	-0.19 (-0.54, 0.17)	-0.19	0.313
No interaction	79.81 (71.29, 88.34)	79.26 (70.73, 87.79)	-0.56 (-5.47, 4.36)	-0.03	0.825
**Lesson time spent *using technology***					
Active use	12.26 (0.74, 23.78)	8.26 (-3.26, 19.78)	4.00 (-8.33, - 0.33)	-0.18	0.070
Passive use	10.74 (4.11, 17.37)	7.04 (0.41, 13.66)	-3.70 (-8.51, 1.10)	-0.24	0.131
No use	76.85 (59.85, 93.85)	84.67 (67.81, 101.81)	7.96 (1.16, 14.78)	0.26	**0.022**

Note: M = Mean; C I = Confidence Interval. Data was adjusted for clustering. Effect sizes were calculated based on means and standard deviations using traditional classroom values as the denominator, these were not adjusted for clustering. Significant differences between traditional classroom and flexible learning spaces (p<0.05)

With respect to the *learning setting*, students in flexible learning spaces spent less class time working as a whole class (d = -0.65, *p* = 0.001), more time working in groups of more than six students (d = 0.46, *p* = 0.004) and in groups of up to six students (d = 0.61, *p* = 0.001) compared with students in traditional classrooms, resulting in moderate effect sizes. The difference in time spent sitting individually between the two conditions was not significant.

Regarding the *modes of learning* category, significant differences were seen in 4 of the 6 behaviour codes (all p = 0.001). Students in flexible learning spaces spent significantly more time collaborating than in traditional classrooms (d = 1.33) resulting in a very large effect. Further students in flexible learning spaces spent less time being engaged in teacher-led instruction (d = -0.75), working independently (d = -0.79) and engaged in presentation-based work (d = 0.65) resulting in moderate to large effect sizes. Difference in time spent engaged in reflective learning and research-based work were non-significant between the two conditions.

With respect to students’ *type of engagement*, students in flexible learning spaces spent significantly more time actively engaged with the lesson content (d = 0.50, *p =* 0.001) and significantly less time verbally off-task (d = -0.44, *p =* 0.016) than in traditional classrooms, resulting in moderate and small effect sizes, respectively. Difference in passive engagement and passive and motor off-task behaviour were non-significant between the two conditions.

In relation to students’ *interaction with peers*, students in flexible learning spaces spent significantly more time interacting positively (d = 0.88, *p* = 0.001) and significantly less time not interacting (d = -0.85, *p* = 0.001) traditional classrooms, resulting in large effect sizes. There was no significant difference in time spent engaged in interactions of a negative nature between the two conditions.

With respect to the proportion of time students spent *using technology*, students in flexible learning spaces spent significantly less time using technology (d = 0.26, *p* = 0.022) than in traditional classrooms, resulting in a small effect size. The differences in time spent using technology both actively and passively, between conditions, was not significant.

## Discussion

This study evaluated differences in student classroom behaviour between traditional classrooms and flexible learning spaces. Students in flexible learning spaces spent significantly less time in a whole-class setting, and more time working in groups, relative to traditional classrooms. In flexible learning spaces, students spent more time collaborating and interacting positively with their peers, as well as more time presenting work back to the class. Further, students spent less time being taught explicitly and working individually, than in traditional classrooms. Overall students in flexible learning spaces spent a greater proportion of class time actively engaged with the lesson. This was demonstrated through verbal and physical behaviours appropriate to the task set by the teacher, such as raising hands, writing or discussing the activity. If a child looked bored, they were still coded as engaged so long as they were doing what was asked of them by the teacher. Students were less likely to be verbally off-task and spent less time engaging with technology relative to students in traditional classrooms.

Student disengagement and lack of motivation are among the key elements that underpin the narrative around why schools are adapting their pedagogical approaches, rethinking the built classroom environment and creating flexible learning spaces [32]. Disengagement is of concern not least because it places students at risk of school dropout [33] but, because low school engagement has been shown to be a correlate and predictor of problem behaviour and poor health among adolescents [34]. On the flip side, fully engaged students report better mental and physical health in addition to improved academic grades [35]. Disengagement has been found to be particularly acute during early adolescence and persists into the secondary school years [36]. While it is recognised that engagement occurs on a cognitive, emotional and behavioural level [37,38], behavioural engagement–which can be classified according to how students interact with the teacher, their peers and the lesson content–is assessed most frequently as it is directly observable [39]. Student engagement is generally categorised and measured as a binary–engaged or disengaged [39]. However, engagement is not constant, but context-specific [37], temporal and thus exists along a continuum [39]. Students often fluctuate multiple times between being actively or passively engaged to being passively, verbally or motor off-task throughout a lesson. This study measured engagement along this continuum and found no differences in time spent off-task (passive or motor) between the two conditions. Instead it was found that students spent a greater proportion of class time actively engaged with the lesson content and less time verbally off-task when in the flexible spaces. Further there was no significant difference in negative interactions among students between the two conditions, but significantly more positive interaction in the flexible learning condition. Since teachers commonly report disruptive behaviour as talking out of turn or disturbing/hindering other students [40], these findings may be reassuring to educators who are concerned about possible challenges around managing disruptive classroom behaviour in these more autonomy-permissive, interactive environments.

Disengagement and detachment from school have been shown to increase as students progress through the grades [41]. Further, an association has been found between middle school instructional environments that increasingly include the whole-class setting and classroom disengagement among youth [41]. In flexible learning spaces students spent a greater proportion of class time working in group settings and collaborating, and were typically given autonomy to interact with one another and discuss academic tasks. It is suggested that this contributed to creating the conditions that fostered the high level of active engagement [42] that were observed. This is supported by findings that suggest that when peer to peer classroom interaction contributes to the creation of a positive interpersonal environment, student engagement increases [43]. It is therefore encouraging to note the greater level of positive interaction among students observed in the flexible learning spaces.

Previous research has demonstrated that a strong student-teacher relationship fosters behavioural engagement [44], and that in classrooms where teachers facilitate dialogue and discussion, student engagement is enhanced [45]. In addition it has been shown that among classroom level factors, teacher-student interactions are the greatest predictor of learning outcomes in standardised tests [46]. While this study did not observe a difference in the proportion of class time spent in teacher-student interaction between the two conditions, the observed interactions that did occur were overwhelmingly positive; i.e., they were related to academic content or rapport building rather than disciplinary in nature. This finding is possibly due to the fact that teachers participating in this study all valued the importance of teacher-student interaction and therefore may have demonstrated greater levels of engagement with their students in both types of conditions, than teachers in traditional classroom typically would.

These outcomes indicate that modifications to the built learning environment of secondary school classrooms, coupled with student-centred pedagogy, can positively influence adolescent behaviour during class time. In this context student-centred is defined as encouraging students to become active participants, engaged in their own learning experiences. This rationale aligns with research in environmental psychology which has long purported that human behaviour and the built environment are closely interrelated [47]. A key difference between these two contrasting learning environments is that in flexible learning spaces teachers actively relinquish their control over where and how students work [5]. This shift in teaching approach, coupled with the affordances of the built environment facilitate student autonomy and engagement with the space and its users. Previous research suggests that students who perceived their teacher to be autonomy-supportive exhibit higher levels of engagement [48]. The student-centred approach allows students to capitalize on opportunities created by the variety of furniture and resources such as the group tables, standing workstations, and writeable walls. Greater interaction and collaboration then flow on from breaking up the whole class setting and creating conditions that foster group work.

It would be simplistic, however, to suggest a linear causal relationship between flexible learning spaces and the outcomes being measured in this study. Rather a complex interplay exists between the built learning environment, the pedagogical approach, the subject being studied, and the student. The findings suggest that a teacher with the environmental competency to maximise the affordances of flexible learning spaces is able to achieve the results found in this study. This has implications for the nature of professional development that is offered to teachers as well as the ongoing support provided at the departmental and local school level, as teachers and students transition into flexible learning spaces. This area is currently underdeveloped [19].

A strength of this study is that the same teacher and students were observed in both conditions. As such, the differences observed in student behaviour can likely be attributed to the changes in the built environment and teaching approach, rather than to differences between cohorts of students. A limitation is that students were only observed on one occasion per school in each of the two conditions, due to the limited time available for researchers to be in the schools. Because this study design is not the gold standard for establishing cause and effect, further experimental research such as randomized trials are needed, to examine the effects of habitual behaviour over longer time frames in flexible learning spaces. Additionally it could be important to investigate the effect of employing a student-centred approach in traditional classrooms since the majority of secondary school classrooms remain traditional and pedagogical changes in themselves may have a beneficial impact on the outcomes measured in this study. This was not measured in the present study as schools were moving from traditional classrooms with teacher-led approaches to flexible learning spaces with student-centred pedagogy, so it was deemed a priority to investigate these two ends of the space/pedagogy spectrum.

Overall these findings add to the limited research from secondary schools that has shown enhanced engagement among students undertaking lessons in innovative learning environments (20). The results suggest that the varied, adaptable nature of flexible learning spaces and the greater use of student-centred pedagogies, facilitate students spending a greater proportion of class time engaging, interacting and collaborating. This may translate into beneficial learning outcomes in the long-term. Further research is required to unpack the complexity of the interplay between the built environment and the pedagogical approaches and how best to support teachers’ environmental competencies to maximise the benefits that flexible learning spaces can offer adolescents.
